# Supplementary material for: PF127 Hydrogel-Based Delivery of Exosomal CTNNB1 from Mesenchymal Stem Cells Induces Osteogenic Differentiation during the Repair of Alveolar Bone Defects
Source: Nanomaterials (Basel). 2023 Mar 16;13(6):1083. doi: 10.3390/nano13061083 (PMC10058633; doi:10.3390/nano13061083)
Supplement: Supplementary file 1 [file nanomaterials-13-01083-s001.zip › nanomaterials-2112405-supplementary.pdf]

nanomaterials

MDPI

Article

PF127 Hydrogel-Based Delivery of Exosomal CTNNB1 from Mesenchymal Stem Cells Induces Osteogenic Differentiation during the Repair of Alveolar Bone Defects

Longlong He [1,2,†], Qin Zhou [1,2,†], Hengwei Zhang [1,†], Ningbo Zhao [1,2] and Lifan Liao [1,2,*]

1 Key Laboratory of Shaanxi Province for Craniofacial Precision Medicine Research, College of Stomatology, Xi'an Jiaotong University, 710004 Xi'an, China
2 Department of Implant Dentistry, College of Stomatology, Xi'an Jiaotong University, 710004 Xi'an, China
* Correspondence: liaolifan2022@126.com; Tel.: +86-15771915456
† These authors contributed equally to this work.

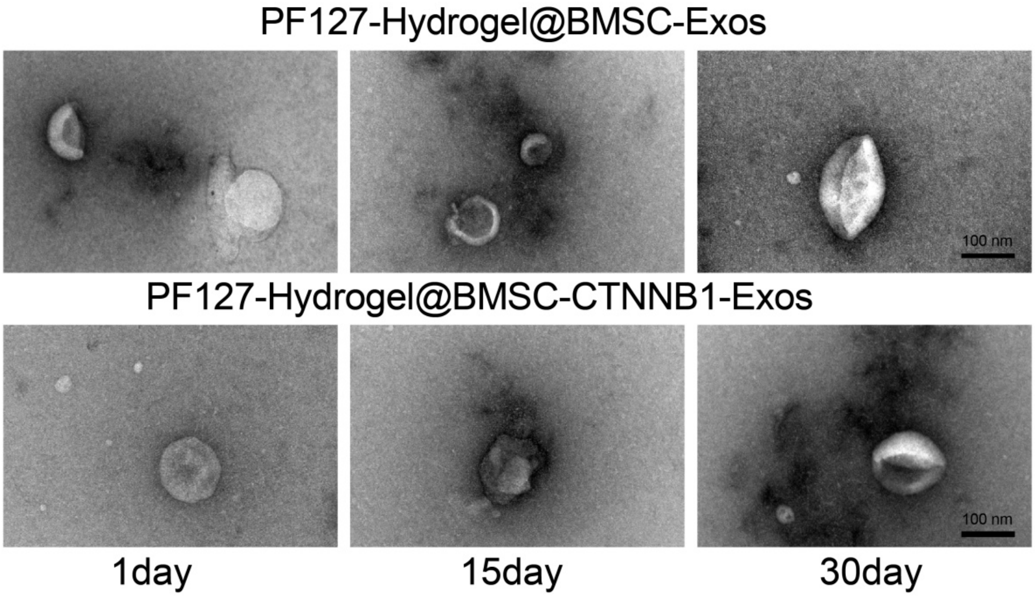

Figure S1. The morphological changes of PF127 hydrogel@BMSC-CTNNB1-Exos and PF127 hydrogel@BMSC-Exos at day 1, 15 and 30 as observed by SEM to test long-term stability.

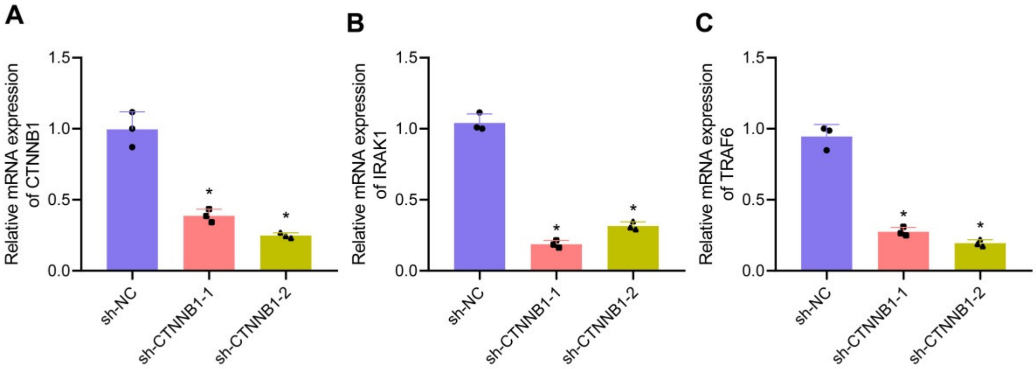

Figure S2. Knockdown efficiency of CTNNB1 (**A**), IRAK1 (**B**) and TRAF6 (**C**) detected by RT-qPCR. * $p < 0.05$ *vs.* the sh-NC group. Cell experiments were repeated three times.

Table S1. Targeted knockdown using shRNA sequences.

shRNA	Sequence
sh-NC	5′-CGATAACGTTACGAAGTACCAT-3′
sh-CTNNB1-1 (rat)	5′-GGGCAATCCTGAGGAAGAAGA-3′
sh-CTNNB1-2 (rat)	5′-GGGCAATCCTGAGGAAGAAGA-3′
sh-IRAK1-1 (rat)	5′-GCAGTAATGAGAAATACTACA-3′
sh-IRAK1-2 (rat)	5′-GGTTTCGTCACCCAAATATCG-3′
sh-TRAF6-1 (rat)	5′-GCGATCGATTGACTGACAACA-3′
sh-TRAF6-2 (rat)	5′-GCTACTATGAGTCTCTTAAAC-3′

Note: sh-, short hairpin RNA; NC, negative control.

Table S2. qPCR primer sequences for ChIP.

• Gene	• Sequence
• p-miR-146a-5p	• Forward: 5′-CTGGCCCCATGTAGAACTGG-3′<br>Reverse: 5′-CCCCCAGGGTACACAAACTC-3′

Note: ChIP, chromatin immunoprecipitation.

Table S3. Primer sequence for RT-qPCR.

Gene	Sequence
CTNNB1 (rat)	Forward: 5′-ATCATTCTGGCCAGTGGTGG-3′
	Reverse: 5′-GACAGCACCTTCAGCACTCT-3′
IRAK1 (rat)	Forward: 5′-CCCCTCCTCCATCAAGCCAAG-3′
	Reverse: 5′-GGTACACACACCCAAAACCAC-3′
TRAF6 (rat)	Forward: 5′-ACTTGATCTCGGAGTGCTGC-3′
	Reverse: 5′-CAGTCAATCGATCGCACACG-3′
RUNX2 (rat)	Forward: 5′-GCGGTGCAAACTTTCTCCAG-3′
	Reverse: 5′-TCACTGCACTGAAGAGGCTG-3′
OCN (BGLAP) (rat)	Forward: 5′-GAATAGACTCCGGCGCTACC-3′
	Reverse: 5′-TCCTGGAAGCCAATGTGGTC-3′
miR-146a-5p (rat)	Forward: 5′-TGAGAACTGAATTCCATGGGTT -3′
	Reverse: Universal sequence
GAPDH (rat)	Forward: 5′-GCATCTTCTTGTGCAGTGCC-3′
	Reverse: 5′-GATGGTGATGGGTTTCCCGT-3′
U6 (rat)	Forward: 5′-CTCGCTTCGGCAGCACA-3′
	Reverse: Universal sequence

Note: RT-qPCR, reverse transcription-quantitative polymerase chain reaction.
